# Danish sperm donors and the ethics of donation and selection

**DOI:** 10.1007/s11019-017-9797-7

**Published:** 2017-09-01

**Authors:** Alison Wheatley

**Affiliations:** Institute of Health & Society, Newcastle University, Floor 2, Newcastle Biomedical Research Building, Campus for Ageing and Vitality, Newcastle upon Tyne, NE4 5PL UK

**Keywords:** Sperm donation, Selective reproduction, Denmark, Personal ethics

## Abstract

There has been a great deal of discussion about the ethical implications of donating sperm and of the ways in which donated tissue is presented, selected, and sold for use in assisted reproduction. Debates have emerged within the academic sphere, from donor offspring and recipients, and in broader popular culture, including questions about the commodification of human tissue and the eugenic potential of selecting donors from particular demographic categories. However, the voices of donors themselves on this subject have been largely silent. This paper draws on data from qualitative interviews with men who donated at a major Danish sperm bank between 2012 and 2013. It argues that many of them are indeed thinking through these complex issues. Donors’ approaches to ethical issues fell into two broad ‘types’: a pragmatic, individualistic approach which focused on more immediate personal consequences, and an ethically-driven approach in which donors considered the impact of donation on offspring and on a wider societal level.

## Introduction

In recent years, there has been a great deal of academic and non-academic attention paid to ethical concerns about the ways in which donor sperm is obtained and used. These concerns have been discussed and debated by law makers and ethical bodies such as the Nuffield Council of Bioethics in the UK or the Danish Ethical Council [*Det Etiske Råd*] in Denmark. These organisations attempt to make sense of the ethical dilemmas that are prominent in this field more generally: namely, what it is acceptable to do in the name of assisting conception of children? This includes questions such as how much information should be available to offspring about donors, whether or not donors should be anonymous, if or how much donors should be paid, and what level of selection is permissible when undertaking donor insemination or IVF. I do not aim to give an answer to these different ethical questions, but rather to illuminate the ways in which the sperm donors in this sample are considering them in their day to day personal experiences of donating. As Erica Haimes (2002: 85) argues, empirical investigation can be used to ‘expand our repertoire of what counts as an ethical’ question by alerting us to the possibility of multiple perspectives on ethics’. I hope to provide a deeper understanding of donors’ perspectives on these issues.

In this paper, I examine the ways in which the sperm donors I interviewed discuss the ethical aspects of their donation, and in particular the uses to which their donation might be put. I consider how donors feel about which and how much information is available about them in online catalogues, and about the recipients’ ability to select donors based on this information. I will argue that donors are considering these ethical issues, but that having personal reservations about the ethics of what they were doing, in terms of the narrow context of the effects on their potential offspring and/or in broader societal terms, was not necessarily a reason for these men to decide against becoming a donor or to stop donating once they had started.

## Sale, selection, and ethical acceptability

Dickenson (2007: 1) writes that ‘it is widely feared that we no longer possess a property in our own bodies.’ The question of ownership has been raised many times in relation to human tissues. Traditionally, English law has assumed that tissue that has been removed from a living body is ‘res nullius’, or belonging to no-one (Quigley 2012). However, many people consider themselves to have a claim on body parts, both from their own bodies and from their families’. Human gametes have only been marketable in their own right for a relatively short time; they were much more difficult to market before the development of technologies that give clinics the ability to isolate specific body parts and tissues for long-term storage and transport. While the European Tissue Directive requires donation be founded on principles of altruism on the part of donors (Council Directive 2004/23/EC 2004), this has not prevented cryobanking from becoming a profitable business.

Resnik (1998) posits that, if we accept a divide between personhood and body, it might be possible to commodify the body without treating a person as a commodity; nothing of one’s personhood is lost by cutting hair, or donating blood. Semen may also fall under this category as men’s bodies constantly replenish the supply, unlike egg donation where each woman’s body has a finite amount of eggs. However, Resnik also suggests that selling gametes may be considered closer to selling a person, since gametes can form a person. This tension between the ease of obtaining of semen and the potential for it to form human life is central to issues around commodification of donor sperm. Holland (2001: 264–5) argues that many people feel a sense of unease at the thought of the ‘billion dollar private-sector industries’ that are based on gametes and other bodily tissues, because they have ‘have an intimate connection to personhood’. “Contested commodities” have multiple and contradictory meanings: ‘internally, we might feel ourselves committed to the notion that the human body is priceless, even as we can wonder what price we might be able to get for the donation of our sperm or eggs’ (*ibid*.: 275). She sees ‘incomplete commodification’ as the answer to the ethical problems of commodifying human tissue, as it allows commodification with regulation. Similarly, Waldby and Mitchell (2006: 137) argue that tissue donation is an example of the entwinement of gift and commodity, where a ‘pure form of either [is] impossible’.

At the sperm bank I visited, the processed and stored straws of semen in the freezers are advertised for sale via online catalogues. The same basic information (race/ethnicity, eye colour, hair colour, height, weight, and occupation) was provided about every donor regardless of type. However, donors could also choose to provide an ‘extended profile’, which was created from a mixture of information that the sperm bank staff record about the donor (including their personal impressions of him) and information that the donor himself provides in the form of a questionnaire. This questionnaire included more in-depth information on the donor’s appearance (such as build, hair texture, and facial shape), family history and health information, and a number of questions about the donor’s personality, hobbies, and experiences. It also included a handwritten message to future offspring; such messages usually contained brief notes about why they had chosen to become a donor. Donors received a larger payment in exchange for submitting one of these extended profiles for display on the website, and recipients paid a surcharge to purchase from donors with extended profiles.

These catalogues are open for the public to browse and all of the information that the donor has allowed to be made available can therefore be viewed before a purchase is made. Potential recipient parents can then buy straws of sperm directly from the sperm bank, according to whichever criteria they prefer to use to select a specific donor. These criteria could be physical appearance, particularly similarity in looks to the recipient parent(s) in order to potentially create family resemblance for bonding purposes or to facilitate non-disclosure of a child’s donor-conceived status; questionnaire responses and donor personality indicators, which allow recipients to pick the donor they think is most ‘suitable’; or any combination of factors based around the information available. The sale of sperm, particularly via the internet, and the potential for recipients to select particular donors has drawn a great deal of media scrutiny, especially with regard to the types of men who are recruited as donors and to non-Danish women travelling to Denmark to obtain Danish sperm (Adrian 2010).

Donor sperm is not an entirely value-neutral product. A number of authors have previously addressed the question of what it is that sperm banks are selling beyond merely selling sperm: in particular, they are selling an idealised masculinity and a particular kind of safe sperm (Moore and Schmidt 1999; Daniels and Golden 2004; Almeling 2007; Kroløkke 2009). Sperm banks have in place a rigorous selection process for donors that encompasses both desirable (or saleable) social and physical traits, and a lack of ‘risky’ behaviour in order to reduce the chances for sperm to carry disease. Therefore, donor sperm is not sold simply as sperm, but rather it is tied closely to the details of the men who provided it. That is to say, buyers are not only buying donor sperm, they are buying donor sperm from a specific donor: sperm that is considered to be imbued with specific qualities based on him, or an idealised version of him. What is unclear from this literature is what donors themselves make of this kind of selective reproductive potential.

This paper draws on data from interviews with donors at a major Danish sperm bank between 2012 and 2013. The Danish context is specifically interesting as a case study for research into sperm donors because of its position as an international hub for donor sperm. A number of UK sperm banks and fertility clinics import Danish sperm due to shortages of sperm from British donors, and both the Danish and British media have been steadily highlighting stories about British people (and other foreigners) travelling to Denmark in order to undergo donor insemination for over a decade. Unlike the UK, where identity-release donation (i.e. donors are required to be open to contact from their offspring when they reach the age of eighteen) is mandatory, Danish donors are able to choose whether or not they wish to donate anonymously or not. This means that recipients who order sperm through the mail for self-insemination without the intervention of a clinic are able to bypass UK laws that forbid the collection and use of anonymous donor sperm.

## Method

This study employed qualitative methods to investigate Danish donors’ experiences of being a sperm donor. The main body of data collection encompassed in-depth semi-structured interviews (see “Appendix”) with donors at several branches of a major Danish sperm bank. The majority (n = 9) of the interviews were carried out face to face, although for privacy and practical reasons, three donors opted to be interviewed via Skype and one via email. The interviews were transcribed as text and thematically analysed with the aid of QSR NVivo 10. Interviews were carried out by a sociologist with a MA in Social Research and informed a PhD study. The research was conducted in accordance with the ethical standards of the University of Edinburgh. Informed consent was obtained from all individual participants. Participants are referred to throughout by a pseudonym to preserve confidentiality.

Participants were recruited initially by means of information leaflets placed in the reception area of the sperm bank, and later via direct email mediated by the sperm bank manager when the initial recruitment method yielded few responses. Out of the 110 donors contacted, a total of 13 donors participated in the study (see Table 1); no information was available on non-respondents. There are a number of possible reasons for the difficulty faced in recruiting. Firstly, the nature of sperm donation as a practice, particularly taking into account the anonymous donors in the sample, may mean men are reluctant to speak about it for fear that they may be ‘outed’ as a donor. Secondly, sperm donation involves a sexual act and is therefore a topic that donors may have felt uncomfortable discussing. It is also possible that this discomfort may have been exacerbated with a female interviewer, though previous research has suggested this is not necessarily the case (e.g. Grenz 2010). Thirdly, conducting interviews in English may have limited the sample pool, and also potentially limited its demographics: we might posit that young, middle-class and/or highly educated Danes would be more likely to meet this criteria. However, the demographics of the eventual sample do not differ broadly from the demographic of the donor base at the time of the fieldwork.

**Table 1:** Participant characteristics

Age	
Median	26.5
Nationality	
Danish	12
Non-Danish	1
Ethnicity	
White	11
Non-white	2
Occupation	
Student	8
Military	2
Other profession	3
Marital status	
Married	4
In a relationship	5
No relationship	4
Children	
Yes	4
No	9

## Information for sale

Ten out of thirteen donors had opted for the extended profile. There was not necessarily a relationship between anonymity and choosing not to provide the extended profile; a mixture of both anonymous and identity-release donors had provided extended profiles and had opted out (see Table 2). Donors who had completed an extended profile generally cited either financial reasons (since providing this profile gave an increase of 10% to their eventual payment), or empathy with the potential recipients or offspring as their reasons, or both. For Lars, an anonymous donor whose motivation for donating was that he needed money, the additional payment was the main factor—he later said that he would not have chosen the extended option otherwise.

**Table 2:** Donation characteristics

Donor anonymity	
Anonymous, extended profile	5
Anonymous, no extended profile	3
Identity release, extended profile	5
Length of donation	
<6 months	1
6 months–3 years	11
>3 years	1
Active donor	
Yes	10
No	3
Other donation	
Blood	1
Blood and organs	4
None	8

Interviewer: Why did you choose [to have an extended profile]?
Lars: Because you got an extra one thousand kroners for an extended profile! [both laugh] (...) As long as I’m doing it, and as long as I have an extended profile, I might as well do as much as I can for the person to make-if of course they are looking at all this information—they can make an informed decision as far as possible, given that I am anonymous. Because if it was me, I would like to know as much as possible about the biological father, to know how my kid might turn out to be [laughs] on some level.

However, having chosen to provide this information, he could empathise with the potential recipients’ desire for it. Giving recipients enough information to make an ‘informed decision’ was also discussed by Andreas, an identity release donor:

Andreas: I believe they know more about me than somebody who has a child with their own husband [laughs] obviously, because I haven’t filled out a questionnaire for my wife. So she doesn’t know everything that’s in the questionnaire, but it’s sort of come in dribs and drabs as she’s known me, you know, so in a sense it’s a very informed decision these people are making. Because it is very, sort of, all round and goes around all kinds of aspects of who I am.

In contrast to Lars, Andreas viewed himself as ‘purely altruistic’; the payment he received from donation was an additional bonus to his main goal of helping people to conceive, as he decided to become a donor after experiencing infertility treatment.

Donors felt that the personal information they provided would be useful to recipients not only in selecting physical donor traits that they hoped would be passed on to their offspring, but also in providing confidence that the donor was someone they could identify with, particularly for anonymous donors whom they could never meet in person. Georg, for example, felt the extended profile and information about personality would be an important factor in his decision making if he were a recipient:

Georg: I think [wanting to choose donors] is a natural thing. If it was my family, so I couldn’t deliver sperm and would have to, to get a sperm donor, I would be very interested in getting the right one, or somebody that, that I could identify with as a father. Also more than just what skin colour and, er, these eye colours and stuff. I would be interested in, like, is it somebody who also is interested in science and all these things. (…) But then I guess that differs from person to person, some people will be more into the, er, how does this person feel and behave, and I would probably be more interested in what interests does this person have, and do they match mine. I think it’s a natural thing.

Some identity release donors had chosen to provide the extended profile because they wanted to offer as much information to their future offspring as possible, even before age of 18:

Interviewer: Do you have an extended profile as well?
Kasper: Oh yes, extended and non-anonymous, because I think, er, looking into the perspective of the child, erm... it must be quite frustrating not to know where your genes stem from. Not that I ever would have a father-son or father-daughter connection with this child. But just to know where your genes stem from, I think that would be quite important to me, if I was this child.

It was less common for donors to talk about providing information for offspring than for recipients, perhaps in part because the majority of the donors were anonymous and did not intend to ever meet their offspring.

## Control over information

Two donors (Christian and Bent) had opted out of extended profiles over concerns around identifiable information being shared online. Bent described feeling uncomfortable with strangers knowing too much about him:

Bent: I honestly don’t remember what people can find out about me. It’s probably my hair colour, that my eyes are blue, my build, er, my profession. But that doesn’t really tell you anything. You get these, stories from America where they can see a picture and they know—they can almost contact your parents if they wanted to, to ask whether you were an easy child or not! And (…) that would be a bit too putting myself out there, slightly Big Brother-like without really being it.

Bent’s account suggests he feels surveilled by recipients (and potentially by others, as the information in the catalogues was openly available) who seem to want to know too much, in contrast to donors such as Andreas who felt it was necessary for an ‘informed decision’. Other donors also had concerns about surveillance; Henrik, though he did have an extended profile, feared donor offspring could use the data available online to track him down:

Henrik: Even though I am anonymous [I was concerned] that some of them could end up finding me. I’ve heard of some cases where they get together and they have the number of the donor. And they can get together and actually find each other, so all the siblings can find each other. And I think there was a case where they started searching on the profile, all the data in it, and found the guy. That has been some concern of mine.

This fear of losing control over whether or not they wanted to meet their potential offspring in the future was common across the sample. Several donors described recent Danish television programmes following donor offspring searching for their biological fathers, and this visibility may well have contributed to these worries about the amount of available information; potential hordes of unanticipated children ‘knocking at the door’ at an unspecified future time were described by Bent and several of the other donors. This fear seemed to stem from the potential to be unexpectedly asked to assume responsibility for the offspring, as well as from their potential to disrupt donors’ family life (Wheatley 2017).

## Ethics of payment

We have seen that payment was an important factor in choosing an extended profile. For some donors, payment was the sole motivating factor for their donation:

Christian: The reality is that 99% of donors are students that need money and don’t care about what happens to their sperm. If donors and parents want to connect of course they should be able to. But this is a well paid job for most people, and nothing else

Indeed, some donors had experimented with abstinence periods and other types of ‘body work’ (such as avoiding alcohol) to help maximise the payment they received, since the sperm bank paid according to quality. Unhappy with this system, Lars had considered switching his labour to a different sperm bank which offered payment at a flat rate:

Lars: Even though on the sites they say they don’t pay for the sperm, they pay for the inconvenience it is for a person to come here, they still pay you according to the amount and quality. So yeah, it’s bogus. [laughs] Because inconvenience is the same for all of us, no matter what the quality is. But the higher quality and the larger the amount, the more you will get paid, and as such, it is of interest to know whether you got 150 kroners or 500, because the margin is quite large. Er, the difference, yeah, in the lowest and highest.
Interviewer: Have you ever had a batch that completely failed?
Lars: Er, yes that happens, sometimes. Erm... that’s just too bad. And again, that’s the bogus part of the saying, of the part where they’re saying “We pay you for the inconvenience”. Yes, you—the batch failed, or not the batch but the donation failed, and that gives me zero kroners, but the inconvenience was still the same as the one that gave me 500.

Here, Lars calls into question what, exactly, is being paid for. In the UK, the Nuffield Council on Bioethics (2011) suggests dividing ‘recompense’ into ‘reimbursement’ of direct financial loss and ‘compensation’ for ‘discomfort’ and other non-financial losses, which Lars’s account suggests are not clearly conceptualised for donors.

For others, there were ethical implications in how much payment donors received and how sale prices were calculated. Georg, for example, had concerns about the final price of sperm being too high for all recipients to be able to access it:

Georg: I’m thinking that the main price on all that are this process like freezing, keeping it, medical stuff and that the actual payments to us donors is a minor thing. And as long as it is like that, then it’s justifiable that we get, these, like, yeah, er... this, what I would think would be a lot of money.

Jonas was in favour of lowering payments, arguing that a level of payment that was too high could induce men to donate who had not fully thought through the ethical implications:

Jonas: [I donate] absolutely because of the money. Absolutely. [But] I would never do it if I couldn’t accept it ethically, if I only saw it as a job I might believe that it was kind of weird. But it’s because I’m fully, I think, I believe myself that I fully understand the consequences of what I’m doing and that’s why I don’t mind doing it.

He drew on a narrative of donor responsibility toward their offspring to inform his definition of an ‘ethical’ donor. This narrative is often used by donor-conceived activists to justify their calls for openness; some donor offspring report feelings of abandonment or of ‘genetic discontinuity’, for example, upon finding out that they were donor-conceived (Turner and Coyle 2000). He did not, however, advocate the full removal of payment for donation; indeed, no donors in the sample did.

## Limitations on donor matching

Selecting based on matching particular physical traits to produce a family resemblance was accepted by the majority of the donors. Some donors, however, were uncomfortable with this selection being made at the level of the individual donor. Jonas had opted out of the extended profile for this reason. He instead suggested that a lottery might be appropriate, matching donors to recipients at random within the bounds of narrow selection criteria:

Interviewer: You mentioned the online profiles and the ability for parents to pick donor characteristics—
Jonas: I don’t like that. I hate that! [laughs]
Interviewer: So how would you rather it was set up?
Jonas: Anonymously! I think that it’s good that there might be a few options to pick off. Something like skin colour and ethnicity, something like that.
Interviewer: Why ethnicity?
Jonas: Only because it can be, it’s not all people that are capable of getting a little black child, for example. If a Southern American got a black child, it would be kind of weird for them I think. It’s not everyone who’s capable of taking that responsibility for something which is basically the same but in some ways culturally are different, right? Which we look at differently in our culture. But basically I think it should be just random, because—well yeah, we’re, it’s just children, right? [laughs]

With this comment, Jonas seems to take the position that ‘children are children’ and recipients should be able to accept a donor for their offspring at random, echoing ideas about the parental imperative to unconditional love (Sandel 2007). However, he also makes allowances for issues of resemblance with regard to ethnicity. An in-depth analysis of ethnicity in sperm banking is beyond the scope of this paper, but the ways in which these categories have been constructed in sperm donation more broadly have been previously documented by others (see e.g. Nordqvist 2012; Hudson 2015). Skin colour is often treated as the most important characteristic to ensure resemblance in donor matching, and it seems therefore unsurprising that donors consider it of utmost importance as well. Parallels could also be drawn between Jonas’s references to cultural difference and narratives of transracial adoption (e.g. Lee 2003).

Although Jonas was the only donor to opt out of the extended profile for reasons of selection, other donors, who had agreed to an extended profile for the extra money, had begun to question their decision. Mikael described his preferred system which would, like Jonas’s, match on broad characteristics of appearance in order to create family resemblance:

Mikael: I think it should be possible to continue the element of chance, so that the donor they eventually receive sperm from will be of the same type as the husband. Well, maybe [laughs] they don’t want that type, but anyway (...) that same general type. So if they want someone that’s 5’10 with curly hair, then give them a donor that’s 5’10 with curly hair, end of story. And not... one out of twenty options in that, erm, in that range.

These suggestions to reduce the granularity of matches seem to at once relate back to concerns around anonymity and surveillance and also to fears about using donor information to make very specific selections to ensure an offspring with certain desired traits.

## Selecting for desired traits

There was a clear boundary of acceptability for donors between matching for resemblance and selecting for traits desired by the recipient parents. Donors such as Bent, for example, articulated a difference between the two:

Bent: I like blondes, my girlfriend’s a blonde, I chose her partly because of that. So, if we couldn’t have a child, I would want a blonde child anyway, probably. Not just because I think it’s more attractive and I want attractive children, but also because people wouldn’t notice it as much if we had a blonde child as if we had a, erm, a brunette or something. So no, I... [sighs] all these ethical questions about choosing your child, it can get a bit too much. I don’t agree with—again, you hear these terrible stories from America where they can almost choose the length of the arms and anything and I wouldn’t want that.

Here, characteristics which would mark a child as a member of the family and avoid potentially difficult questions from outsiders, such as hair colour, are acceptable, whereas characteristics that are less likely to contribute to family resemblance, such as arm length, are not. This idea of ‘choosing your child’ was discussed negatively by several donors, reflecting wider debates about the ethics of creating ‘designer babies’, particularly as it relates to the ethics of selecting for disease- versus non-disease-related traits (Sterckx et al. 2013). Mikael, for example, explicitly illustrated a dichotomy between selection for health, which he argued was for the benefit of society, and selection for appearance:

Mikael: The thing is that selective breeding really isn’t something that humans should do. In my mind, if it ruined dogs, which it did, then it will probably ruin Homo Sapiens as well. (…) What annoys me about it is probably the fact that people might be choosing out of the wrong reasons. Some of the reasons that would make sense to me would be (pause) I think I was told, er, a while ago that, for some reason, Scandinavians, at least Swedes, Norwegians and Danes, have a mutation on our T-cells that makes us slightly more immune to HIV.^1^ And if that, if that is actually true, then that would make sense, because it’s something that makes the human race better. On the other hand, when people start saying “yeah! we want Scandinavians because they’re tall, and blonde, and blue-eyed” then (…) I start to worry, because that’s the wrong way of looking at it.

The emphasis on tall, blonde and blue-eyed here seems to echo fears around eugenics and the value of particular sets of physical traits (Daniels and Golden 2004); there is a sense that the particular traits of Danish donors that might be desired by recipient parents are tied to social desirability and prejudice.

Mikael further argued that that the amount of information available about donors was excessive in comparison with norms of partner selection:

Mikael: I don’t like the idea of people having too many things to choose from when choosing a donor. Because they might not choose from the same things when they choose a partner, and that’s the point really. I mean, when we meet somebody we don’t really know how they are, how it would be like to live with them. And that process takes a long time and [is] kind of short-circuited when you choose a sperm donor, because it’s basically just a catalogue you choose from. So that part kind of makes me uncomfortable.

He believed that it was wrong for recipients of donor sperm to choose based on an ‘ideal’ partner rather than the characteristics of their actual partner. In contrast, Isak argued that the selection potential of donor insemination reflected other forms of selective reproduction already available.

Isak: I don’t think it’s a good idea that you can like choose the eye colour of your children or the hair colour, but you know what, a lot of people get an abortion if the child has Down’s Syndrome, I mean... we are already in the process of selecting some children and throwing out others. And... of course, I’m part of that selection process but then again, I think it’s more up to the society to discuss what is right and wrong, and I like the fact that parents have the opportunity to select as they would have in adoption settings. I mean, if they have a preference of a child from Ethiopia because of some cultural thing instead of a kid from Asia, I don’t know... erm... that’s the same kind of selection for me.

Isak drew on ideas about abortion and disability to argue that there is already selection taking place in society; the incidence of Down’s Syndrome in Denmark, for example, has been falling since a new pre-natal screening policy was introduced in 2004 (Ekelund et al. 2008). There is potential, then, for donors to be drawing on debates outside of the sphere of donor insemination in particular to inform their personal ethics around the topic as well as drawing on the donation debates themselves. Isak connects his place in the process of selective reproduction with ethics at the societal level.

Jonas emphasised that he would be willing to disclose personal details to his potential offspring but described selection for desirable traits as ‘problematic’:

Jonas: So what I would most prefer to be in would be the closed personal profile so you don’t know anything about me, but not anonymous, so the children would have the opportunity to look me up 1 day, if that’s what they wanted to do.
Interviewer: Why would you prefer it that way round?
Jonas: I think it’s very problematic, the trend that is going on right now (…) a lot of technology is focusing on how to create babies with DNAs, like, I want a blonde baby, I want a darker, I want a smart baby because I only want the ones that have been to university, I want a beautiful child, and I don’t like that. I think that’s very problematic in many ways. But I still think that it’s important that—we’ve got to accept it’s not a natural thing, it’s not normal to give birth through a sperm donor.

It is clear, therefore, that not all donors who were in favour of reducing opportunities for selection were in favour of not collecting that information at all; rather, they believed that limitations should be put in place on who could access information and for what purpose.

## Discussion

In becoming a sperm donor, the men in this study were obliged to make choices regarding openness and the provision of information: the decision to remain anonymous or to become an ‘identity release’ donor open to contact with offspring when they come of age; and, separately, the decision to complete an extended profile for the donor catalogue. Donors often drew on moral narratives when discussing their reasons for making particular choices, invoking ideas about selective reproduction in the process.

‘Matching’ donors’ physical characteristics to those of recipient parents is established practice in gamete donation, historically because maximising the resemblance between the child and the recipient father allows heterosexual couples to hide the stigma of male infertility (Daniels and Taylor 1993). However, family resemblance also plays a role in the formation of kinship bonds by helping ‘locate’ the child in the family group (Hargreaves 2006: 273), including in the context of lesbian conception (Nordqvist 2010). In contrast, the ‘designer baby’, a child who through active selection or direct genetic modification is born with particular traits, has been the subject of a great deal of ethical controversy (see e.g. Fauser and Devroey 2011). Designer babies represent a combination of ‘biogenetic control, consumer demand, and parental desire’ (Franklin and Roberts 2006: 1), seeming to contravene norms that would position all children as ‘gifts or blessings’ (Sandel 2007). Opinion has often been split regarding the acceptability of selecting out disease-related traits and non-disease-related traits (Sterckx et al. 2013). The donors in the sample also differed on the extent to which they considered selection wrong. While most donors were in favour of matching for family resemblance, some were against any characteristics other than ethnicity being used while others were in favour of other markers of physical resemblance such as hair colour. Others felt that physical characteristics were ‘trivial’ but suggested that selecting for desirable health-based traits, such as HIV resistance, was acceptable for reasons of public health. Several donors argued that selecting for desirable characteristics was morally wrong—but although many donors spoke at length about their ethical concerns regarding selection, these questions and ethical misapprehensions were not enough to prevent them from donating.

Many of the donor narratives involve the ‘naturalisation’ of selection and technological mediation in some way. Naturalisation is defined by Cussins (1998) in her study of infertility clinics as

the rendering of states of affairs and facts in a scientific or biological idiom, and the means by which certain uncertainties, questionings, and contingencies are rendered unproblematic, “natural”, or self-evident.

This second idea is evident in accounts of donation from participants. Donors who are questioning the ethics of issues such as selection in assisted reproduction can draw on pre-existing narratives of ethics in other activities that are considered to be ‘natural’, such as mate selection, or already ‘naturalised’, in the case of adoption. The question of selection and the comparison of ‘mate choice’ with sperm donation has been made in the past by authors who suggest that women value the same kind of physical appearance and personal characteristics in both their selection of sperm donors and of long-term romantic partners (Scheib et al. 1997). This kind of naturalisation narrative allows the donors to render unproblematic the ethics of this particular facet of donation. On the other hand, as some donors argued, the amount of information available to potential donor sperm recipients was much greater than the amount of information available in a ‘natural’ mate selection situation. Therefore, donors can use or resist the ‘naturalisation’ of donation in order to support their personal ethics.

At the beginning of this paper I quoted Erica Haimes, who argues that it is possible for empirical research to give us multiple perspectives on ethics to help us to consider what an ‘ethical’ question might actually be. In the discussion that followed, I have explored the kinds of ethical questions that donors are thinking about and using to inform their decisions about whether and how to donate. I have focused broadly on questions about the availability of information and the potential for selective reproduction, though these were not the only ethical issues that donors were considering: the questions of how much payment should be given for sperm donation, how many children each donor should be able to produce, and whether or not anonymous donation should be permitted were also discussed by donors. There is no single viewpoint on these questions across all of the donors in this sample, or across donors of a particular ‘type’ (e.g. anonymous donors). Rather, there seem to be two main approaches. The first is typified by a pragmatic, individualistic approach to issues of payment and selection in decision making as it relates to donation. Donors of this approach believed that donors should be paid and viewed donation as a job, perhaps investing time in achieving the most money. They had concerns about becoming identifiable but may have chosen to have an extended online profile due to the financial incentive. Their attitude towards selection was one of empathy with the recipient parents: they felt that they would want the same information if he was in the position of having to choose a sperm donor. The second approach might be considered ethically-driven. Donors of this approach made decisions based strongly on the (perceived) needs and desires of the potential donor offspring. They were in favour of donors being identity-release, but also wanted tighter restrictions on the information available to potential recipients prior to donation due to concerns about the morality of selective reproduction. They viewed donors who saw their donation as ‘just a job’ as irresponsible, but also placed responsibility for ethical decisions around selection on wider society.

While there were also donors who had not spent time contemplating their personal ethical conception of what they were doing, many of the donors had thought about and expressed opinions on issues such as selection, anonymity, and payment. This is in contrast to popular images of sperm donors as indifferent to the context of their donation beyond the immediate monetary reward (Almeling 2011: 143; Thomson 2008). The ethical questions that donors are considering are not necessarily the same ones that have been most important in the academic and media debates: all were content with the status quo of the choice between anonymous and identity-release donation, for example. The main ethical question was around the amount of personal information available to recipients, either as part of a set of wider concerns around the effects of selection, or because the donors were concerned for their own future well-being. Therefore, the ‘personal ethics’ that the donors have vary in scale: some donors are concerned about the effect of their actions on them and those close to them, some on the donor recipients, some on the donor offspring, and some on a societal level.

## Footnotes

1 The allele CCR5-delta32 has been shown to be responsible for certain types of HIV resistance, and is primarily found in Nordic populations (Lucotte 2001).
